# Editorial Correspondence

**Published:** 1880-06

**Authors:** 


### EDITORIAL CORRESPONDENCE.

*Dr. J. G. Westmoreland, Atlanta, Ga:*

MY DEAR SIR—I have been a constant reader of the Atlanta MEDICAL AND SURGICAL JOURNAL since my graduation in medicine five years ago and must state that I have obtained more sound practice from it than any other Journal that has come under my observation. I write to you for information respecting a malady, which prevails in our county, sometimes to an alarming extent, and hope that you may see proper to tender the services required—I speak of "Malarial Hæmorragic Fever." Will you please be so kind as to write an article on the subject, for the July number of the JOURNAL, or make special mention of the prathological condition observed by physicians, who have been favored with an opportunity of examining the bodies of those who succumbed to the disease.

If engagements are such that you can't grant the request, will you please refer the matter to some able correspondent, whose location has furnished him with such material for study? Our County Medical Association has appointed this subject for discussion at its August meeting, and we are anxious to obtain all the practical bearings, from competent men, that can be had.

Yours Truly, G. W. BASKETT.

With pleasure response is made to the enquiries and suggestions of our esteemed correspondent.

The nature and treatment of primary lesions in the various forms of "the fever" have been the subjects of discussion for the past two centuries. Until fifty years ago the medical profession generally were in utter ignorance of the organ primarily affected by the poison. The stomach, liver, spleen, bowels and the blood were, each, from time to time and by different authors, set down as the point first disturbed directly.

The compiled lectures of Stokes & Bell, published in Europe some forty-five years ago, promulgated the true pathological lesion in fevers.

For the first time in my hearing this pathology was taught in a regular didactic course of lectures by Dr. L. D. Ford, of Augusta, in 1843. Medical men were of course slow to accept the doctrine, and not until the last twenty-five years has the opinion of spinal disturbance in malarial fever, been generally adopted by physicians. Few now can be found who do not endorse this theory, and use remedies applicable to this pathological condition. Difference of opinion exists as to the particular varieties of "the fever" not all of which are strictly speaking, malarial. They are known as simple intermittent, congestive or malignant intermittent, remittent or bilious, yellow, hœmaturial, dengue or broke-bone typho-malarial or two weeks fever, typhoid, typhus and scarlet fever. All agree that the first three are of malarial origin and noncommunicable from one person to another. In the general tendency and symptoms, however, these differ from each other decidedly. While simple intermittent fever has no violent or alarming symptoms and may be cured in a day, the congestive or malignant form gives immediate alarm and is exceedingly dangerous to life. Remittent fever differs materially from these and, though sometimes proving serious, rarely terminates fatally under the present improved plan of treatment.

Though differing so widely from each other these forms occur at the same time and place, to all appearance, in the same kind of subjects, and are controlled by the same general remedy.

The malarial origin of dengue and hæmatarial fever, I

believe, is generally admitted, and yet they are not generally relieved by quinine. The former rarely requires decided treatment, subsiding in a few days, while the latter frequently resists every effort at cure.

Yellow fever is believed to be malarial by some, among whom I have been for sometime recorded. This, when malignant, defies all forms of treatment usually, and differs from hæmaturial fever, not in color of the skin nor the hæmorrhagic tendency, but in the particular part from which the exuding of blood occurs. Black vomit in yellow fever is evidently the result of hemorrhage into the stomach while in the other, the blood proceeds from the urinary organs. In both diseases the hemorrhage is probably the result of a lower degree of vitality or nervous power, from the excessively depressed condition of the spinal functions. Virulent and abundant malaria may speedily so effectually destroy nervous power as to produce black vomit, while hæmaturia is generally the result of slow malarial poisoning from long exposure and frequent attacks of the usual forms of malarial fever. Lowered vitality leads to hemorrhage from these parts, perhaps, as it does in the latter stage of typhoid fever from the bowels. The hemorrhage has no immediate connection with the primary pathological condition, but is an evidence or result of that degree of nervous prostration from which it is difficult to revive. Remedies in all such cases to stay the loss of blood, are of course incidentally demanded, but can not reach the prime trouble. Nervous power must be restored and supported, else dissolution must ensue. This is of course difficult to accomplish, when the poisoning has gone to the extent of destroying the susceptibility to neurotic stimulants and tonics.

Of the remedies used in these or any other form of malignant malarial fever, chloroform, opium, alcohol, quinine and spinal counter-irritants, have, perhaps, afforded all the permanent benefit that has been had from treatment.

In seeking new remedies for these fevers it is therefore only necessary that we find something to whose action the nervous centers are more susceptible than those just mentioned.

To counteract the immediate hemorrhagic tendency I have reason to believe carbolic acid will prove highly useful. In the dose of two to five or six grains repeated every three or four hours the blood is made more coagulable, and hemorroage, in consequence, will likely be, to some extent, at least, controlled. This suggestion is made without having tested it in this particular form of hemorhage, but we feel confident of benefit from it.

Hæmaturial fever, unfortunately, generally is developed in persons whose systems are broken down by frequent attacks of fever, in whom the blood, as well as the tissues, has lost its proper quality and functions, from the long continuance of nervous inactivity from the effect of malarial poison on the spinal center. So depraved and inactive the organs become that the nervous system is doubtless not susceptible to the ordinary action of remedies in extreme cases. Under such circumstances, success with any plan of treatment need not be expected.

This is evidently the case in malignant yellow fever. Every plan of treatment will fail when the nervous system is so oppressed that it is not susceptible to the action of medicines.

Having my attention called to the subject of fevers by the above correspondence, I am reminded of a communication received sometime since from a physician of North Carolina, in which the writer, alluding to an article in this Journal, on mushroom fever, as it prevailed in 1847 and in 1879, expresses a desire that I should write more fully in regard to treatment.

While it is supposed by some, and perhaps correctly, that marsh malaria consists of cryptogamous spores, it is certainly an essentsally different variety of poison from that of the highland mushroom. Fever produced by the latter has very little tendency to intermissions or remissions, and, instead of seven days, the usual duration of remittent fever, it rarely if ever ceases under two weeks. Owing to the more continued form of this than marsh malarial fever the inference may be justly drawn that the poison attacks a portion of the center higher in the spinal column or within the cranium itself. Typhoid fever resembles this in most if not all respects, except the duration, and as there is reason to believe the medulla suffers most in typhoid, it seems reasonable that typhomalarial is the result of primary disturbance in the same neighborhood.

Blisters to the back of the head and neck seem to give more relief from the violent symptoms than any other treatment; and until the delirium and high febrile action has been thus controlled, quinine is useless to say the least of it. Some cases, however, soon slide into an intermittent state, which requires quinine and opium only to make a favorable termination of the disease, and a speedy getting up after the fourteen days of its duration.

